# Platelet hyperreactivity and frailty in a mouse model of Alzheimer’s disease are prevented by anti-oxidant treatment

**DOI:** 10.1007/s11357-025-01710-w

**Published:** 2025-06-03

**Authors:** Mauro Vismara, Silvia Maria Grazia Trivigno, Marta Zarà, Stefania Momi, Paolo Gresele, Marina Camera, Ilaria Canobbio, Gianni Francesco Guidetti, Mauro Torti

**Affiliations:** 1 Department of Biology and Biotechnology L. Spallanzani, University of Pavia, Via Bassi 21, 27100 Pavia, Italy; 2 University School for Advanced Studies IUSS, Pavia, Italy; 3 Department of Medicine and Surgery, University of Perugia, Perugia, Italy; 4 Centro Cardiologico Monzino IRCCS, Milan, Italy; 5 Department of Pharmaceutical Sciences, University of Milan, Milan, Italy

**Keywords:** Frailty, Alzheimer disease, Platelet reactivity, Tempol, Anti-oxidant treatment

## Abstract

Frailty is an age-related syndrome commonly associated with different comorbidities, and its occurrence is particularly frequent in patients with Alzheimer’s disease (AD). A persisting low-grade inflammation has been suggested to favor the onset of both AD and frailty. Besides their role in hemostasis and thrombosis, blood platelets are true inflammatory cells, and their direct contribution to the onset and progression of AD has been documented. In this work, we investigated whether platelet hyperreactivity and pro-oxidative functions are implicated in the development of frailty in a mouse model of AD, the APP23 mice. Assessment of 31 specific clinical signs of deterioration in mice at 3, 9, and 18 months of age demonstrated that the development of frailty was significantly more pronounced in the APP23 mice compared to wild-type littermates. In 18-month-old APP23 mice, a significant platelet hyperreactivity was detected as shown by a significantly stronger platelet aggregation in response to submaximal stimulation of both collagen and thrombin receptors. Moreover, the pro-inflammatory function of platelets, evaluated as circulating and agonist-induced platelet-neutrophil aggregate formation, was significantly increased in aged APP23 mice compared to wild-type littermates. Platelet hyperreactivity was partially prevented by prolonged treatment with the anti-oxidant agent Tempol, which reduced both agonist-induced aggregation and platelet-neutrophil aggregate formation. Importantly, prolonged treatment of APP23 mice with Tempol significantly reduced also the frailty index score in 18-month-old animals. These results outline the possible beneficial effect of an anti-oxidant treatment in hampering platelet hyperreactivity and preventing the onset of frailty associated to AD.

**Supplementary Information:**

The online version contains supplementary material available at 10.1007/s11357-025-01710-w.

## Introduction

Frailty represents an age-associated condition characterized by excessive vulnerability to external stressors consequent to the reduction of physiological reserves and causes a decline in multiple physiological systems [1]. Frailty is a major challenge in geriatrics as it negatively impacts life expectancy, with frail people having a higher risk of adverse health outcomes and premature death. A recent meta-analysis of population-level studies reported an overall frailty prevalence of 17% in people aged > 50, which increases to 45% for pre-frailty, an intermediate state between fitness and frailty [2]. In both cases, prevalence is higher in women than in men, possibly because of a combination of genetic, epigenetic, and psychosocial factors [2, 3].

The biological events and conditions determining whether an aged individual remains fit or develops frailty are unknown, and the different outcomes of the physiological aging process are likely to involve a combination of genetic, biological, pathological, and environmental factors [4]. However, a growing number of studies have provided robust evidence that chronic low-grade inflammation and oxidative stress are associated with the development of frailty [5, 6]. Importantly, circulating markers of inflammatory reactions and of unbalanced redox conditions are typically increased in frail patients [7]. Cell damage caused by persistent oxidative stress triggers inflammatory reactions, which in turn alter redox balance and generate a vicious circle that gives rise to a condition of unresolved and persisting chronic low-grade inflammation. This chronic, low-grade inflammation that often develops during aging is provoked by a continuous antigenic load and associated with a reduced efficacy of the anti-inflammatory mechanisms and has been termed “inflammaging.” Inflammaging progressively weakens the body’s ability to adequately respond to external injuries and thus contributes to the pathogenesis of age-related diseases [8].

Macrophages, as well as circulating leukocytes, are directly involved in inflammaging. However, in the last decades, a robust body of evidence has documented that also blood platelets play a central role in this process [9]. Besides their fundamental role in hemostasis and thrombosis, platelets are now considered true inflammatory cells [10]. Upon activation, blood platelets release a number of pro-inflammatory cytokines and produce reactive oxygen species (ROS) able to alter the redox balance. Moreover, activated platelets interact with neutrophils and monocytes to stimulate specific pro-inflammatory responses. The possible contribution of platelets in the onset of frailty has not been considered so far.

It is well documented that chronic low-grade inflammation and unbalanced redox conditions that predispose to frailty are also associated with age-related diseases, such as diabetes, cardiovascular disease, and Alzheimer’s disease (AD) [11–13]. AD is the most common form of dementia affecting over 50 million individuals worldwide and is characterized by the accumulation in the brain of senile plaques, made of the amyloid peptides (Aβ1–40 and Aβ1–42) deriving from the amyloidogenic processing of the membrane amyloid precursor protein (APP) [14, 15]. The pathophysiology of AD is still poorly understood, but growing evidence points to a role of blood platelets in the onset and progression of AD. Platelets express high levels of APP and are the main source of circulating amyloid peptides [16]. Moreover, platelets display pro-inflammatory and pro-oxidative actions, thus participating in the evolution of a low-grade inflammatory state that favors the onset of AD [17].

Interestingly, a robust bidirectional correlation between AD and frailty has been documented. Frailty prevalence in patients with mild to moderate AD is very high, ranging from 11.1 to 50.0% and averaging at 31.9% [18]. Conversely, frailty itself accelerates cognitive decline in AD patients [19].

In this study, we used a well-characterized mouse model of AD, the transgenic APP23 mice, to investigate the development of frailty in the context of AD. Transgenic APP23 mice express the human gene for APP751 bearing the Swedish mutation (K670 N/M671L) under the control of the neuronal promoter Thy1. APP23 mice have a sevenfold increased expression of mutant human APP and develop extensive senile plaques in brain parenchyma starting from 6 months of age [20]. Mice also develop cerebral amyloid angiopathy at a later age (12 months) [21]. Although none of the available mouse models represents the perfect model for reliable translation of results to human disease, due to both genetic and epigenetic factors as well as the multifactorial nature of the pathology [22], the APP23 model presents some peculiar advantages, including the ability to develop cerebral amyloid angiopathy and the fact that disease signs appear relatively early.

The concomitant development of frailty in aged APP23 mice has never been investigated. The Aβ amyloidogenic peptides that accumulate in aged APP23 mice are agonists for blood platelets promoting secretion and aggregation [23, 24]. Additionally, amyloidogenic peptides induce platelet-mediated oxidative stress and pro-inflammatory reactions, which may predispose to frailty development [25]. Therefore, we also investigated whether long-term treatment with anti-oxidant drugs may ameliorate the onset of frailty in the context of AD by reducing the pro-inflammatory activity of circulating platelets. Our results demonstrate that the frailty index in adult and aged APP23 mice can be significantly reduced by long-term administration of Tempol, a cell-permeable free radical scavenger able to control some pro-inflammatory activities of blood cells.

## Materials and methods

### Materials

AYPGKF (TRAP4) was synthesized by DBA. Convulxin was provided by Dr. K. J. Clemetson, (Theodore Kocher Institute, University of Berne, Switzerland). PE-conjugated JON/A antibody against the active conformation of integrin αIIbβ3 was from Emfret Analytics. BV421-CD62P and BV510-CD41 were obtained from BD Biosciences. PE-Ly6G/Ly6C and PerCP-CD45 were from eBioscience. Apyrase, prostaglandin E1 (PGE1), and 4-hydroxy-2,2,6,6-tetramethylpiperidinooxy (Tempol) were from Sigma-Aldrich.

### Animal models and treatments

APP23 transgenic mice in C57BL6 J/Rcc background were kindly provided by Dr. Diego Albani, IRCCS-Istituto di Ricerche Farmacologiche “Mario Negri,” Milan. Colonies were bred at the animal facility of the University of Pavia, to generate both APP23 transgenic mice for the study and control wild-type littermates. All the procedures involving the use of wild-type and transgenic mice were approved by the Ethics Committee of the University of Pavia and the Italian Ministry of Public Health (authorization number 19/2020 PR).

For this study, three groups of mice of both genotypes (wild type and APP23) were used: young mice at 3 months of age, adult mice at 9 months of age, and aged mice at 18 months of age. Inter-individual variation in the age of animals used at the time of experimental observation was not longer than 1 week. At the age of 3 months, each group of APP23 mice was further divided into two groups: untreated and treated with Tempol. At least 10 mice for each group were maintained. Tempol was administered daily to two subgroups of APP23 mice at the final concentration of 5 mM in drinking water, while the other two subgroups received only vehicle. Considering an average assumption of 3 ml/day of water, mice were treated daily with about 90 mg/kg of Tempol. All the groups of mice (wild type and APP23) at 3, 9, or 18 months were subjected to clinical observations for quantification of frailty and then sacrificed for blood collection and analysis of platelet function, as described below. The effect of long-term treatment with a low dose of Tempol was analyzed in the subgroups of APP23 mice at 9 and 18 months of age, thus 6 or 15 months after the starting of the treatment. During this period of time, no evident side effects associated with Tempol treatment were noticed.

### Quantification of frailty

The quantification of frailty was assessed using a 31-item frailty index, based on established clinical signs of deterioration in mice, as outlined by Whitehead J.C. et al. [26]. The clinical assessment encompassed the evaluation of multiple physiological parameters, including the integumentary, musculoskeletal, vestibulocochlear/auditory, ocular, nasal, digestive, urogenital, and respiratory systems, as well as signs of discomfort, body weight (g), and body surface temperature (°C). Table 1 details the clinical signs of deterioration and deficits evaluated in this study. Mice were initially observed in their home cages and subsequently transferred to an assessment room. Each mouse was weighed, and body surface temperature was measured using an infrared temperature probe directed at the abdomen, with the mean value obtained from three readings. Subsequently, mice underwent a brief clinical examination to assess the parameters listed in Table 1. The severity of each deficit was rated on a standardized scale: a score of 0 indicated no deficit, 0.5 indicated a mild deficit, and 1 indicated a severe deficit. Deficits in body weight and body surface temperature were scored relative to reference values established for control mice (wild-type littermates). Aside from the infrared temperature probe, the only additional equipment utilized was a clicker, similar to those used in dog training, for auditory assessment. The assessment of frailty could not be performed in double-blind; however, frailty parameters were determined by three individual observers, and the frailty index was calculated by averaging their independent evaluations.

**Table 1:** **Thirty-one-item clinical assessment for frailty index score in mice**. The table lists the 31 items used for clinical assessment and the assigned score to calculate the frailty index score in mice

Parameter	Clinical assessment of deficit	Scoring
Integument		
Alopecia	Gently restrain the animal and inspect it for signs of fur loss	0 = normal fur density
		0.5 = < 25% fur loss
		1 = > 25% fur loss
Loss of fur colour	Note any change in fur colour from black to grey or brown	0 = normal colour
		0.5 = focal grey/brown changes
		1 = grey/brown fur throughout body
Dermatitis	Document skin lesions	0 = absent
		0.5 = focal lesions (e.g. neck, flanks, under chin)
		1 = widespread or multifocal lesions
Loss of whiskers	Inspect the animal for signs of a reduction in the number of whiskers	0 = no loss
		0.5 = reduced number of whiskers
		1 = absence of whiskers
Coat condition	Inspect the animal for signs of poor grooming	0 = smooth, sleek, shiny coat
		0.5 = coat is slightly ruffled
		1 = unkempt and un-groomed, matted appearance
Physical/musculoskeletal		
Tumours	Observe the mice to look for symmetry. Hold the base of the tail and manually examine mice for visible or palpable tumours	0 = absent
		0.5 = < 1.0 cm
		1 = > 1.0 cm or multiple smaller tumours
Distended abdomen	Hold the mouse vertically by the base of their tail and tip backwards over your hand. Excess fluid visible as a bulge below the rib cage	0 = absent
		0.5 = slight bulge
		1 = abdomen clearly distended
Kyphosis	Inspect the mouse for curvature of the spine or hunched posture. Run your fingers down both sides of the spine to detect abnormalities	0 = absent
		0.5 = mild curvature
		1 = clear evidence of hunched posture
Tail stiffening	Grasp the base of the tail with one hand, and stroke the tail with a finger of the other hand. The tail should wrap freely around the finger when mouse is relaxed	0 = no stiffening
		0.5 = tail responsive but does not curl
		1 = tail completely unresponsive
Gait disorders	Observe the freely moving animal to detect abnormalities such as hopping, wobbling, circling, wide stance, and weakness	0 = no abnormality
		0.5 = abnormal gait but animal can still walk
		1 = marked abnormality, impairs ability to move
Tremor	Observe the freely moving animal to detect tremor, both at rest and when the animal is trying to climb up an incline	0 = no tremor
		0.5 = slight tremor
		1 = marked tremor; animal cannot climb
Forelimb grip strength	Hold the mouse. Allow it to grip the bars on the cage lid. Lift animal by the base of the tail to assess grip strength	0 = sustained grip
		0.5 = reduction in grip strength
		1 = no grip strength, no resistance
Body condition score	Place mouse on flat surface, hold tail base, and manually assess the flesh/fat that covers the sacroiliac region (back and pubic bones)	0 = bones palpable, not prominent
		0.5 = bones prominent or barely felt
		1 = bones very prominent or not felt due to obesity
Vestibulocochlear/auditory		
Vestibular disturbance	Hold the mouse by the base of the tail and lower it onto a flat surface. Observe for any tilting, rotation, or bending of the head, or twisting of the torso	0 = absent
		0.5 = mild head tilt and/or slight spin when lowered
		1 = severe disequilibrium
Hearing loss	Test the startle reflex. Hold a dog training clicker approximately 10 cm from the mouse and activate it three times, recording the response	0 = always reacts (3/3 times)
		0.5 = reacts 1/3 or 2/3 times
		1 = unresponsive (0/3 times)
Cataracts	Visually inspect the mouse for the presence of any opacity in the central area of the eye	0 = no cataract
		0.5 = small opaque spot
		1 = clear evidence of opaque lens
Eye discharge/swelling	Visually inspect the mouse for any signs of eye discharge or swelling	0 = normal
		0.5 = slight swelling and/or secretions
		1 = obvious bulging and/or secretions
Microphthalmia	Inspect the eyes for abnormalities	0 = normal size
		0.5 = one or both eyes slightly small or sunken
		1 = one or both eyes very small or sunken
Corneal opacity	Visual inspection of the mouse to superficial white spots and/or clouding of the cornea	0 = normal
		0.5 = minimal changes in cornea
		1 = marked clouding and/or spotting of cornea
Vision loss	Lower mouse towards a flat surface. Evaluate the height at which the mouse reaches towards the surface	0 = reaches > 8 cm above surface
		0.5 = reaches 2–8 cm above surface
		1 = reaches < 2 cm above surface
Menace reflex	Move an object towards the mouse’s face 3 times. Record whether the mouse blinks in response	0 = always responds
		0.5 = no response to 1 or 2 approaches
		1 = no response to 3 approaches
Nasal discharge	Visual inspection of the mouse to detect nasal discharge	0 = no discharge
		0.5 = small amount of discharge
		1 = obvious discharge, both nares
Digestive/urogenital		
Malocclusions	Grasp the mouse by the neck scruff, and invert and expose teeth. Look for uneven, overgrown teeth	0 = mandibular longer than maxillary incisors
		0.5 = teeth slightly uneven
		1 = teeth very uneven and overgrown
Rectal prolapse	Grasp the mouse by the base of the tail to detect signs of rectal prolapse	0 = no prolapse
		0.5 = small amount of rectum visible below tail
		1 = rectum clearly visible below tail
Vaginal/uterine/penile prolapse	Grasp the mouse by the base of the tail to detect signs of vaginal/uterine or penile prolapse	0 = no prolapsed
		0.5 = small amount of prolapsed tissue visible
		1 = prolapsed tissue clearly visible
Diarrhoea	Grasp the mouse and invert it to check for signs of diarrhoea. Also look for fecal smearing in home cage	0 = none
		0.5 = some feces or bedding near rectum
		1 = feces plus blood and bedding near rectum, home cage smearing
Respiratory		
Breathing rate/depth	Observe the animal. Note the rate and depth of breathing as well as any gasping behaviour	0 = normal
		0.5 = modest change in breathing rate and/or depth
		1 = marked changes in rate/depth, gasping
Discomfort		
Mouse grimace scale	Note facial signs of discomfort: (1) orbital tightening, (2) nose bulge, (3) cheek bulge, (4) ear position (drawn back), or (5) whisker change (either backward or forward)	0 = no signs present
		0.5 = 1 or 2 signs present
		1 = 3 or more signs present
Piloerection	Observe the animal and look for signs of piloerection, in particular on the back of the neck	0 = no piloerection
		0.5 = involves fur at base of neck only 1 = widespread piloerection
Other		
Temperature	Measure surface body temperature with an infrared thermometer directed at the abdomen (average of 3 measures). Compare with reference values from sex-matched adult animals	0 = differs by < 1 SD from reference value
		0.25 = differs by 1 SD
		0.5 = differs by 2 SD
		0.75 = differs by 3 SD
		1 = differs by > 3 SD
Weight	Weigh the mouse. Compare with reference values from sex-matched adult animals	0 = differs by < 1 SD from reference value
		0.25 = differs by 1 SD
		0.5 = differs by 2 SD
		0.75 = differs by 3 SD
		1 = differs by > 3 SD

### Preparation of washed platelets

Mouse blood was collected using a mix of ACD (152 mM sodium citrate, 130 mM citric acid, and 112 mM glucose) and 3.8% sodium citrate (2:1) as anticoagulant, and platelets were isolated essentially as previously described [27]. Briefly, whole blood was diluted in Tyrode’s buffer (10 mM HEPES, 137 mM NaCl, 2.9 mM KCl, 12 mM NaHCO3, pH 7.4) and centrifuged at 120 × *g* for 10 min to obtain platelet-rich plasma (PRP). Apyrase (0.02 units/ml) and PGE1 (1 μM) were then added to PRP before centrifugation at 550 × *g* for 8 min. The platelet-poor supernatant was discarded, and the platelet pellet was washed once with PIPES buffer (136 mM NaCl, 20 mM PIPES; pH 6.5) and then resuspended in Tyrode’s buffer. Purified platelets were maintained at 37 °C in the presence of 5.5 mM glucose, 1 mM CaCl_2_, and 0.5 mM MgCl_2_ unless otherwise indicated.

### Analysis of platelet aggregation and integrin activation

The aggregation of murine-washed platelets was analyzed in a light transmission aggregometer (Chrono-Log Corporation). Washed platelets from wild-type or APP23 mice, at the final concentration of 2.5 × 10^8^ cells/ml, were incubated at 37 °C in the presence of 1 mM CaCl_2_ and then stimulated under constant stirring with TRAP4 or convulxin. Agonists were used at variable low concentrations able to elicit reproducible submaximal aggregation of platelets obtained in different preparations over the 18 months of the study. The following range of concentrations was used: TRAP4 0.25–0.5 mM and convulxin 25–50 ng/ml. Aggregation was monitored continuously for 5 min and quantified using the AGGRO/LINK software.

Analysis of integrin αIIbβ3 activation in resting and stimulated platelets was performed by flow cytometry measuring the binding of the JON/A antibody, specific for the active form of murine integrin αIIbβ3, as previously described [26]. Briefly, samples were prepared by mixing heparinized (10 units/ml) whole blood, Tyrode’s buffer containing 2.5 mM CaCl_2_, and PE-conjugated JON/A antibody. Platelets were left untreated or stimulated with TRAP4 (0.25–0.5 mM) or convulxin (25–50 ng/ml) in the dark for 10 min at 37 °C. The reaction was blocked by adding 0.5% PFA, and samples were analyzed by flow cytometry using the BD FACS Lyric instrument (BD Biosciences) at the PASS-BioMed facility of the University of Pavia (Italy). Data were analyzed using FlowJo software.

### Measurements of platelet-neutrophil aggregates

For the analysis of platelet-neutrophil aggregates, 22.5 µl of whole blood in 3.8% sodium citrate was stimulated with TRAP4 (0.25–0.5 mM) or convulxin (25–50 ng/ml) for 10 min, fixed with 1% PFA in PBS and incubated for 30 min with BV510-labeled anti-CD41 and PerCP-conjugated anti-CD45 antibodies. For neutrophil identification, anti-Ly6G/Ly6C antibody was used. Neutrophils were identified based on their morphology and positivity for CD45 and Ly6G/Ly6C. Platelet-neutrophil aggregates were quantified as percent of neutrophils positive for the platelet antigen CD41 on their surface.

### Data analysis and statistics

Data are presented as either means ± S.D. of at least seven independent experiments from different blood mouse samples. Statistical analyses were performed using GraphPad Prism software (version 10.2.3). Two independent groups were compared using a *t*-test, whereas multiple comparisons were made using one-way ANOVA with Tukey’s post hoc test after verifying the normal distribution of the data. All tests were two-tailed, and a value of *p* < 0.05 was considered to be statistically significant.

## Results

### Frailty development in APP23 mice

APP23 transgenic and genotype-matched wild-type control mice were bred at the animal facility of the University of Pavia and monitored over a period ranging from 3 to 18 months of age. The development of frailty was analyzed by adopting the noninvasive method described by Whitehead et al. [26] with some modifications, as detailed in the “Materials and methods” section. A summary of the parameters analyzed and of the score assigned is reported in Table 1.

**Fig. 1:**
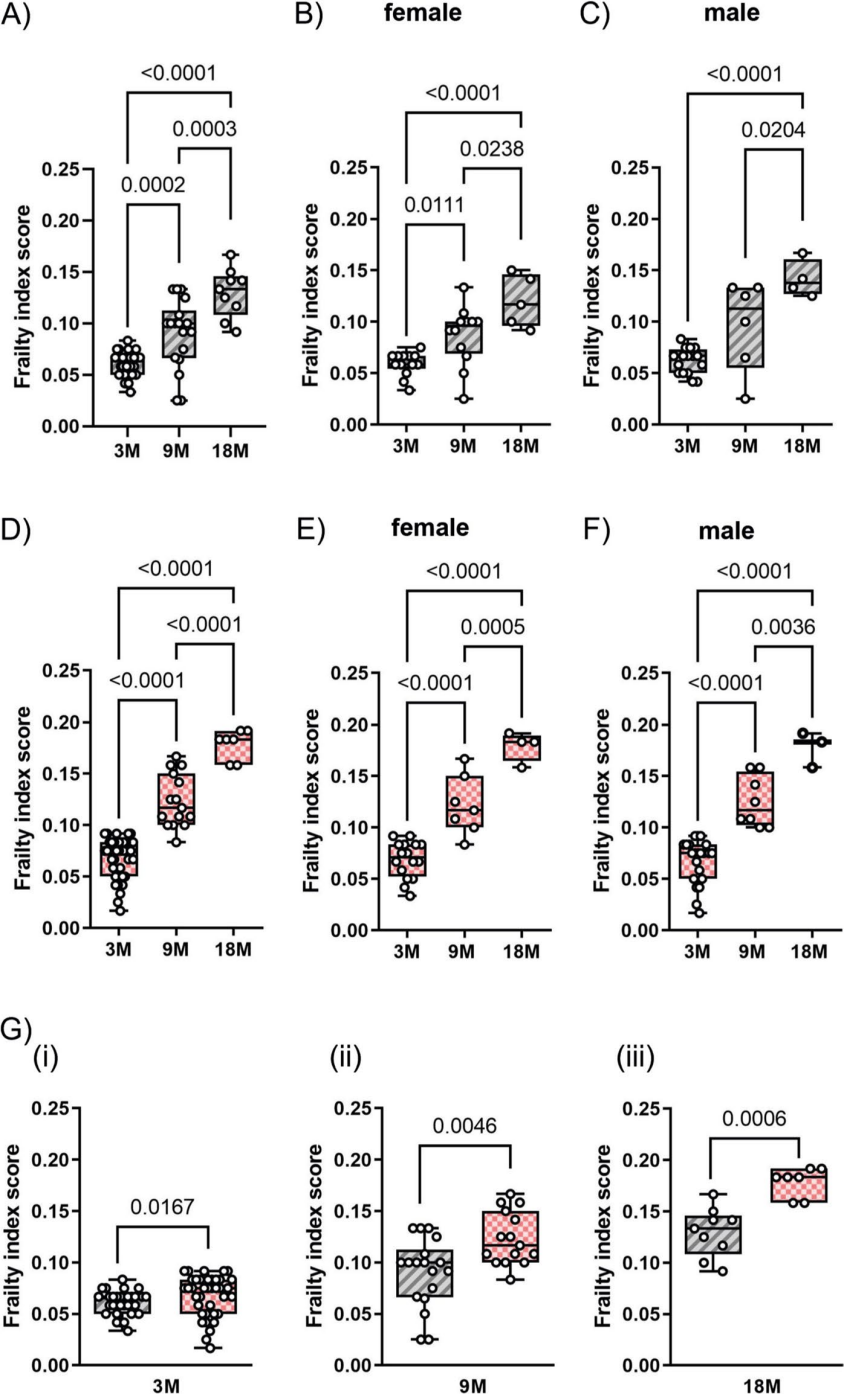
**Frailty index score increases with age in WT and APP23 mice**. Frailty index scores were obtained using the 31-item frailty index evaluation. WT and APP23 mice are represented in grey and in pink, respectively. The age of the mice is expressed in months (3 M: 3 months, 9 M: 9 months, 18 M: 18 months). **A**, **D**. The frailty index scores of WT and APP23 mice, respectively. For both genotypes, the scores were separately analyzed in females and males (**B**, **C** for WT mice; **E**, **F** for APP23 mice). **G**. A direct comparison of frailty index scores between WT and APP23 mice of different ages. Data are presented in box-and-whisker plots, showing the median and distribution values for at least 7 mice. Data shown in (**A**–**F**) and (**G**(i–iii)) were analyzed by one-way ANOVA and *t*-test, respectively. Exact *p*-values are reported

We found that the calculated frailty index score significantly increased in wild-type mice at 9 and 18 months of age compared to young animals (3 month old) (Fig. 1A). At 18 months of age, the calculated frailty index (0.13 ± 0.023) was comparable to that reported by Whitehead et al. [26] for the old group of animals (19 months of age, 0.12 ± 0.008), thus validating our application of the proposed methodology.

Within the groups of animals of the same age, we experienced a high variability of the measured frailty index with a consequent increase in the standard error of the mean values. Nevertheless, the higher frailty index scores in mice of 9 and 18 months of age resulted in statistically significant differences when compared to that of younger animals. Interestingly, when compared to the young animals, the frailty index score measured at 9 months of age was significantly higher only in females, but not in male mice (Fig. 1B, C), supporting the notion that females are more prone to develop frailty [3].

To evaluate the development of frailty in association with AD, we calculated the frailty index score of APP23 mice at 3, 9, and 18 months of age. As shown in Fig. 1D, the frailty score increased significantly over time in these mice. Compared to wild-type animals, we observed a reduced variation of the measured frailty indexes among animals of the same group of age and, as a consequence, a reduced standard error and a higher statistical significance among the observed differences. Moreover, in contrast to wild-type mice, the frailty index score in APP23 mice at 9 months of age was significantly higher than that of younger animals not only in females, but also in males (Fig. 1E, F). A direct comparison demonstrated that the development of frailty was slightly but significantly more pronounced in APP23 mice compared to wild-type littermates at 3 (panel i), 9 (panel ii) and at 18 (panel iii) months of age (Fig. 1G).

### Treatment with Tempol reduces the onset of frailty in APP23 mice

**Fig. 2:**
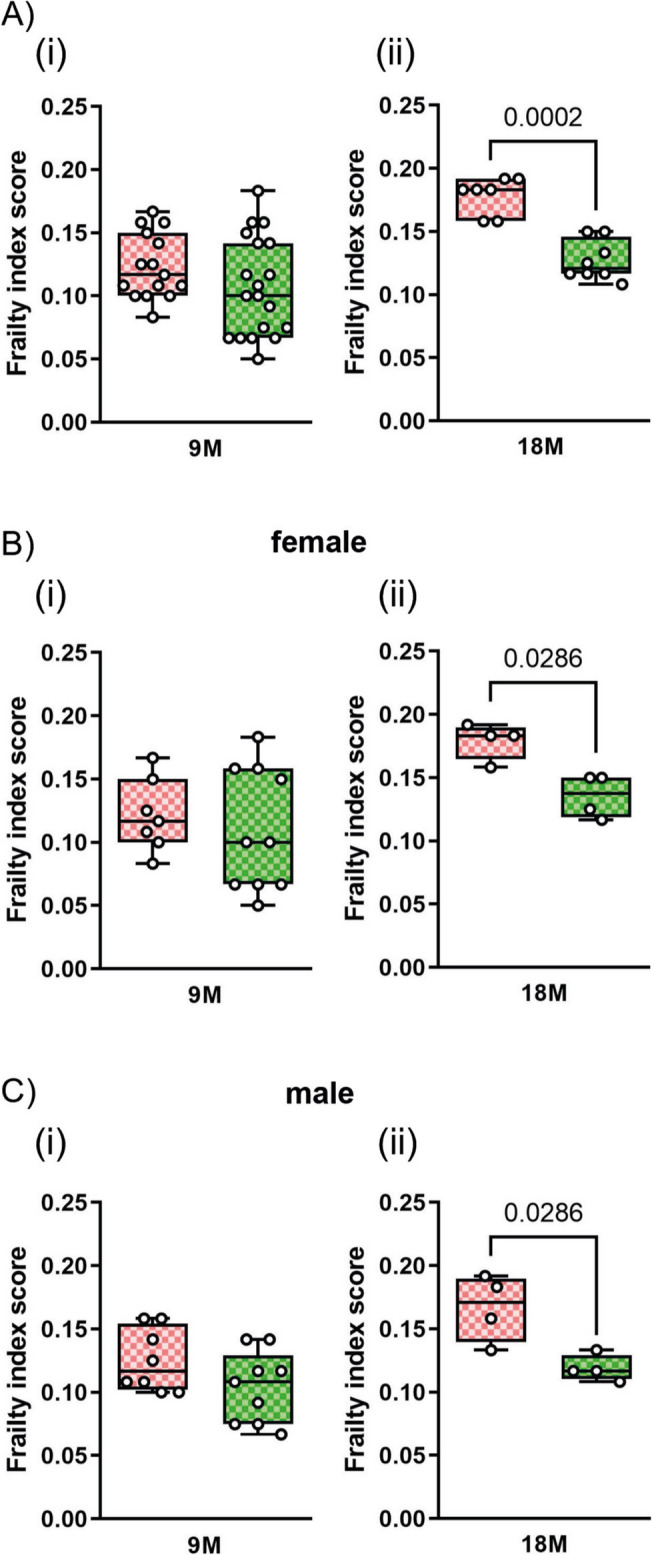
**Tempol reduces the frailty index score in aged APP23 mice**. Frailty index scores were assessed using a 31-item evaluation. Data for untreated APP23 mice are reported in pink, while data for Tempol-treated APP23 mice are reported in green. The age of the mice is indicated at the bottom of the charts. Comparison of frailty index scores between untreated and Tempol-treated APP23 mice is shown in (**A**(i, ii)). Scores were also analyzed separately for females (**B**(i, ii)) and males (**C**(i, ii)). Data are presented as box and whisker plots, showing the median and distribution values for at least 7 mice. Data shown in (**A**–**C**) were analyzed by *t*-test, and exact *p*-values are reported

We next evaluated whether persistent low-grade oxidative stress could favor the development of frailty in APP23 mice. To this purpose, the anti-oxidant agent Tempol was dissolved in the drinking water at the concentration of 5 mM and administered daily to APP23 mice starting at the age of 3 months. Considering an average assumption of 3 ml/day of water, mice were treated daily with about 90 mg/kg of Tempol. The frailty index score evaluated on animals at 9 months of age (thus after 6 months of treatment) was comparable in mice receiving Tempol versus non-treated animals (Fig. 2A(i)). However, at 18 months of age, the prolonged treatment with Tempol (15 months) significantly reduced the development of frailty in the APP23 mice (Fig. 2A(ii)). This effect at 18 months of age was evident both in male and female mice (Fig. 2B(ii), C(ii)). Specifically, in Tempol-treated animals, the frailty index score at 18 months was comparable to that measured at 9 months (0.10 ± 0.039 at 9 months versus 0.12 ± 0.015 at 18 months), indicating that the treatment with an anti-oxidative agent prevented the worsening of the frailty development.

### Effect of Tempol on blood platelet aggregation

Prolonged administration of the anti-oxidative agent Tempol is expected to reduce a wide range of oxidative reactions in treated animals. In order to get insights into the possible mechanism of the protective action of Tempol on the onset of frailty, we explored the possibility that this agent could affect the pro-thrombotic and pro-inflammatory reactions triggered by blood platelet activation. We thus compared platelet function and responsiveness in untreated and Tempol-treated APP23 mice.

We first analyzed platelet aggregation induced by two different agonists able to operate distinct signaling pathways: the peptide TRAP4, a specific agonist of the thrombin receptor PAR4 that induces platelet activation through a G-protein-mediated mechanism, and the snake venom convulxin, a specific agonist of the collagen receptor GPVI that promotes platelet activation through the stimulation of a protein tyrosine kinase cascade. In all cases, agonists were used in a low range of concentrations able to elicit submaximal responses, as indicated in the “Materials and methods” section. Representative platelet aggregation traces are reported in Supplementary Fig. 1 (panels A to E).

**Fig. 3:**
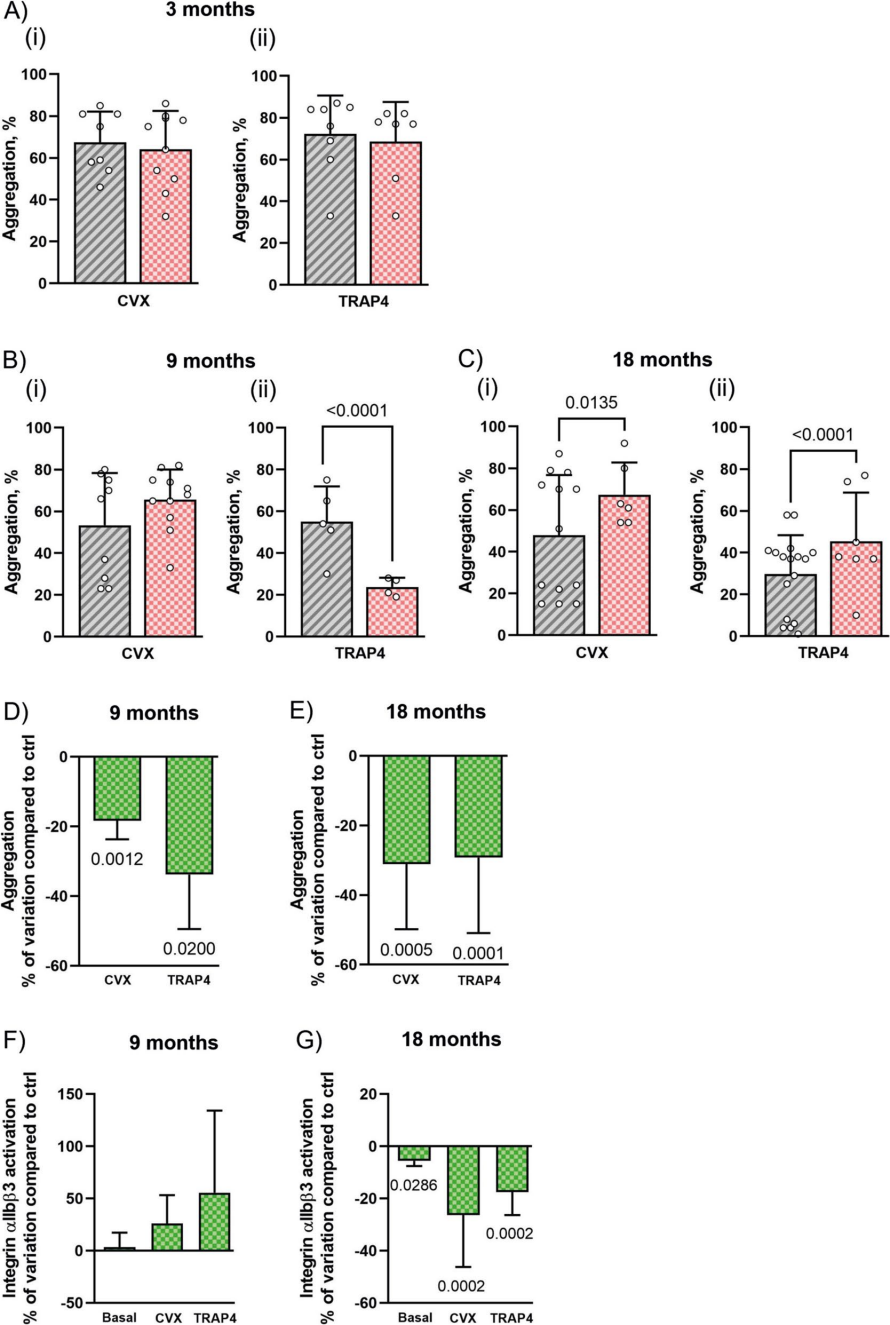
**Tempol attenuates hyperactivity in platelets from APP23 mice**. Washed platelets (2.5 × 10^8^ cells/ml) from WT and APP23 mice were stimulated with convulxin (25–50 ng/ml CVX) or TRAP4 (0.25–0.5 mM), and aggregation was monitored for 5 min using a light transmission aggregometry. WT and APP23 mice are represented by grey and pink bars, respectively. Maximal aggregation (%) is reported for 3-month-old mice (**A**(i, ii)), 9-month-old mice (**B**(i, ii)), and 18-month-old mice (**C**(i, ii)). **D**,** E**. The effect of treatment with Tempol on platelet aggregation in APP23 mice of 9 and 18 months of age. Data are reported as the percentage of variation of aggregation in Tempol-treated APP23 platelets compared to control, where control is the aggregation of platelets from untreated animals of the same age stimulated with the same agonist (convulxin or TRAP4), as indicated on the bottom. The effect of treatment of Tempol on integrin αIIbβ3 activation in APP23 platelets of 9 or 18 months of age is reported in (**F**) and (**G**), respectively. Data are reported as the percentage of variation of integrin αIIbβ3 activation in Tempol-treated APP23 platelets compared to the activation measured in platelets from untreated animals of the same age, unstimulated (basal) or stimulated with convulxin or TRAP4, as indicated. Results are presented as mean ± SD from at least 4 independent experiments. Data shown in (**A**–**C**) were analyzed by *t*-test, and exact *p*-values are reported

We initially compared the reactivity of platelets isolated from wild-type and APP23 mice at 3, 9, and 18 months of age. Figure 3A(i, ii) shows that, in young animals, platelet aggregation was comparable between wild-type and APP23 mice. In 9-month-old adult APP23 mice, we observed a reduced platelet aggregation in response to TRAP4 (Fig. 3B(ii)) but not to convulxin (Fig. 3B(i)). By contrast, in adult mice of 18 months of age, aggregation in response to both agonists was significantly stronger in APP23 compared to wild-type mice (Fig. 3C(i, ii)). This observation is consistent with the notion that platelet hyperreactivity is common in mouse models of AD and may play a role in the onset of the pathology [17, 28, 29].

The treatment of APP23 mice with Tempol since the age of 3 months led to an important impairment of agonist-induced platelet aggregation. At 9 months of age (Fig. 3D), platelet aggregation was slightly but significantly reduced in response to all the agonists analyzed, and this effect was even more pronounced at 18 months (Fig. 3E).

Platelet aggregation is consequent to agonist-induced activation of integrin αIIbβ3 which acts as a fibrinogen receptor. We measured integrin αIIbβ3 activation by flow cytometry after stimulation with convulxin or TRAP4 for 10 min in order to evoke maximal response. Representative flow cytometry histograms are reported in Supplementary Fig. 1 (panels F and G). We found that the reduced platelet aggregation in aged APP23 mice treated with Tempol was actually associated with a defective activation of integrin αIIbβ3. This effect, however, was detectable and statistically significant mainly in mice of 18 months of age (Fig. 3G), but not at 9 months (Fig. 3F).

Platelet activation is also associated with granule secretion. We compared agonist-induced secretion of α-granules by measuring P-selectin exposure in wild-type and APP23 mice of different ages, but we failed to detect any significant difference (data not shown). Moreover, in aged APP23 mice, long-term treatment with Tempol did not affect P-selectin exposure (data not shown). These results indicate that, unlike aggregation, platelet secretion is not altered in APP23 mice and is not affected by anti-oxidant treatment. These results show that defective platelet aggregation is mainly consequent to impaired integrin αIIbβ3 activation rather than reduced secretion.

### Tempol reduces the formation of platelet-neutrophil aggregates

**Fig. 4:**
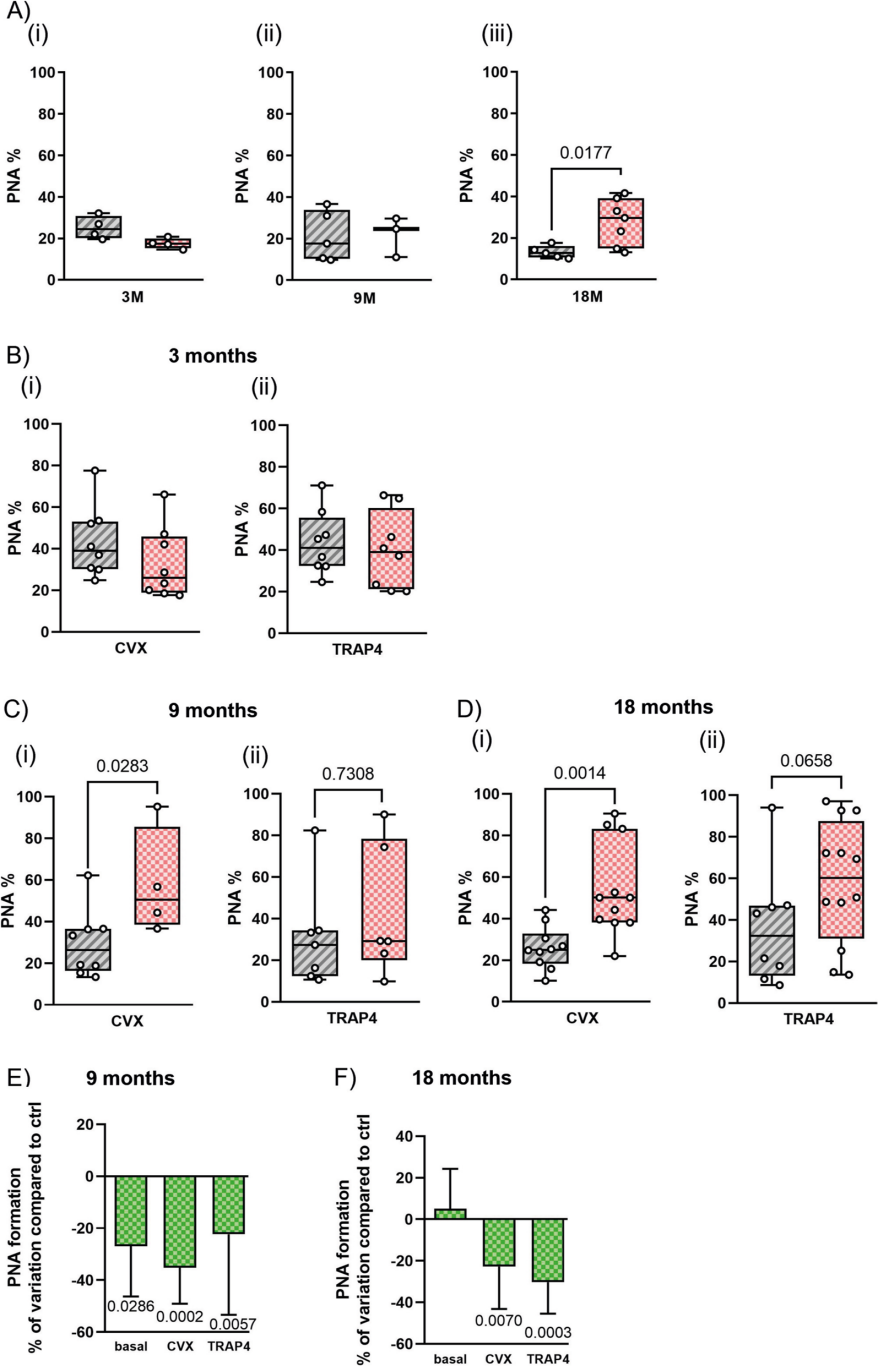
**Tempol reduces platelet-neutrophil aggregate (PNA) formation in APP23 mice under basal and stimulated conditions**. **A**. The percentage of PNA present in whole blood from WT (grey bars) and APP23 mice (pink bars) at 3 (i), 9 (ii), and 18 (iii) months of age was assessed by flow cytometry. **B**–**D**. Platelets in whole blood from WT (grey bars) and APP23 mice (pink bar) at 3 (i), 9 (ii), and 18 (iii) months of age, as indicated on the top of the panels, were stimulated with convulxin (CVX) or TRAP4 as indicated on the bottom. The formation of PNA was then assessed by flow cytometry. **E**,** F**. Effect of treatment with Tempol on PNA formation in APP23 mice. Platelets in whole blood from control and Tempol-treated APP23 mice at 9 and 18 months of age, as indicated on the top of the panels, were left untreated (basal) or stimulated with convulxin (25–50 ng/ml) or TRAP4 (0.1–0.5 mM). PNA formation was determined by flow cytometry. Results are reported as % of neutrophils associated to platelets. The variations of PNA formation in samples from Tempol-treated animals are compared to equally stimulated samples from control, untreated animals. For all the panels, data represent the mean ± SD from at least 4 independent experiments. Data shown in (**A**–**F**) were analyzed by *t*-test, and exact *p*-values are reported

In order to more specifically investigate the pro-inflammatory function of blood platelets, we measured the formation of platelet-neutrophil aggregates (PNAs) by flow cytometry. Representative plots are reported in Supplementary Fig. 2A. A comparison of the percentage of PNA detectable in the circulating blood from wild-type and APP23 mice at 3 or 9 months of age failed to reveal any significant difference (Fig. 4A(i, ii), respectively). However, in 18-month-old adult mice, the amount of circulating PNA was significantly higher in APP23 compared to wild-type mice, indicating a more pronounced pro-inflammatory behavior of platelets in the AD mouse model (Fig. 4A(iii)). Since platelets were found to be hyperactive in the APP23 mice, we next analyzed the ability of stimulated platelets to form aggregates with neutrophils. Platelet agonists TRAP4 and convulxin were added to whole blood from WT and APP23 mice, and the formation of PNA was evaluated by flow cytometry. Figure 4B shows that in young mice of 3 months of age, convulxin (panel i) and TRAP (panel ii) induced the formation of a comparable amount of PNA in wild-type and APP23 mice. However, at 9 and 18 months of age (Fig. 4C, D, respectively), platelet stimulation triggered the formation of a significantly higher amount of PNA in APP23 mice compared to wild-type controls (see Supplementary Fig. 2B, C, and D for representative plots). We tried to compare the PNA formation between males and females across both genotypes at 9 and 18 months of age. However, the sample size was insufficient to support a reliable statistical analysis to draw conclusions about sex-based differences (data not shown). These results suggest that hyperreactivity of platelets in APP23 mice is associated with the development of a stronger pro-inflammatory interaction. To verify the possible beneficial effect of a prolonged anti-oxidant treatment, we analyzed the formation of PNA in the APP23 mice group treated with Tempol since the age of 3 months. Figure 4E shows a small reduction of the amount of PNA detected in whole blood of Tempol-treated APP23 mice at 9 months of age that, however, was no longer evident at 18 months of age (Fig. 4F). However, the ability of TRAP4- or convulxin-stimulated platelets to form aggregates with neutrophils was significantly reduced both at 9 (Fig. 4E) and at 18 months (Fig. 4F) of age (see Supplementary Fig. 3 for representative plots). The reduction of PNA formation was comparable when platelets were stimulated with convulxin or TRAP4 (Fig. 4E, F). These results indicate that the anti-oxidant agent Tempol reduces platelet reactivity and limits the formation of PNA in aged APP23 mice.

## Discussion

In the aged population, frailty represents a general functional decline that dramatically compromises the quality of life. The mechanism for the development of frailty is largely unknown, but it has been clearly demonstrated that the onset of frailty is more common in the context of other age-related diseases, such as diabetes and AD [1]. Our previous work has demonstrated a multifactorial link between AD, blood platelets, and oxidative stress [17]. The Aβ peptides are strong platelet agonists and stimulate the oxidative and pro-inflammatory function of these cells through NADPH oxidase–mediated ROS production. These effects have been proposed to contribute to the progression of AD [23–25, 30].

In the present study, we provide evidence that the pro-inflammatory action of blood platelets can also contribute to the onset of AD-associated frailty. We have demonstrated that Tempol, an anti-oxidant agent and ROS scavenger, affects the pro-thrombotic and pro-inflammatory activities of blood platelets and prevents the development of frailty in a murine model of AD. Our results support the notion that pharmacological targeting of oxidative stress–triggered low-grade inflammation may be beneficial in preserving functional organ homeostasis during aging.

We have performed a longitudinal study using a well-characterized AD murine model, the APP23 mice, that have been followed for 18 months to monitor frailty development and platelet reactivity in comparison with WT healthy littermates. The possibility to evaluate frailty in mice has been validated by several previous studies and provides a strategy that allows a deep investigation on the mechanism underlying this condition [26, 31]. Different approaches for frailty evaluation in mice have been described. In our work, we have adopted the protocol proposed by Whitehead et al. [26], based on the noninvasive assessment of physical parameters that allowed us to subsequently proceed with blood withdrawal and analysis of platelet function.

A major finding of the present study is that the development of frailty is more frequent in the APP23 mice than in control littermates, both at 9 and 18 months of age. Interestingly, male and female APP23 mice are equally sensitive to develop frailty, while, among WT animals, frailty had a more rapid onset in females rather than in males. The development of frailty in AD mouse models has been previously addressed by a few studies. In the 5xFAD mice, expressing human APP and PS1 genes carrying three and two mutations, respectively, the frailty index increased at 11 months of age compared to three 3-month-old animals and was associated with higher mortality [32]. Moreover, using a combination of physical parameters observation and locomotor function analysis, Kapphan et al. documented that the frailty index in 16-month-old 5xFAD mice was higher than that observed in control littermates of the same age [33]. Interestingly, female mice showed a milder phenotype compared to males. Frailty was also investigated in the 3xTG mice, carrying mutations on the APP, PS1, and Tau proteins. The assessment of the frailty index, calculated according to the methods of Whitehead et al., indicated that aged male 3xTG mice develop stronger frailty compared to control WT animals, as well as female 3xTG mice [34]. Despite the observation of a correlation between frailty development and higher mortality, none of these studies addressed the mechanisms for frailty development or tried to identify treatments able to prevent it.

In this context, the present study moves a significant step ahead. We have extended the analysis of frailty to the APP23 mouse model that, differently from other mouse models previously studied, also develops cerebral amyloid angiopathy (CAA) [20, 21] and thus appears more suited to investigate the impact of vascular processes on AD and frailty. Importantly, this model has been previously used to demonstrate the contribution of blood platelets to the development of CAA and AD [28, 35]. It is of note that, in these mice, the amyloid deposition in the brain, as well as spatial memory defects in Morris Water Maze, were already detected at 3 months of age and reported to increase afterwards [20, 36]. This indicates that an altered phenotype is temporally associated with the increased development of frailty detected both at 9 and at 18 months of age.

We have demonstrated that the higher frailty index measured in APP23 mice compared to WT littermates was correlated with a stronger platelet reactivity evidenced by a stronger aggregation in response to different types of agonists and a more pronounced tendency to interact with neutrophils. This was particularly evident in mice at 18 months of age. Aggregation of platelets in 9-month-old APP23 mice was comparable to that of control littermates (or even reduced, as for instance in response to PAR4 stimulation) despite the frailty index being higher. This observation suggests that platelet hyperreactivity is functionally related to the progression of frailty rather than to its onset. This conclusion is also supported by the observation that inhibition of platelet oxidative functions by Tempol did not alter the frailty index at 9 months of age but prevented its progression at 18 months of age. Importantly, Tempol reduces platelet reactivity in terms of both aggregation and interaction with leukocytes, already in 9-month-old mice, and thus, it is plausible that this constitutive platelet inhibition over the following months prevents the progression of frailty.

Tempol is a membrane-permeable cyclic nitroxide that acts as a radical scavenger and superoxide dismutase-mimetic [37]. Tempol has been widely used as an anti-oxidant agent in cell biology studies, but it is also considered a potential pharmaceutical tool [38]. As an anti-oxidant agent, Tempol can limit the formation of reactive oxygen species and, in various experimental models of inflammation and shock, displays neuroprotective, anti-inflammatory, and analgesic effects. Tempol is in a phase 3 clinical trial for the treatment of acute respiratory diseases, and it has also been demonstrated to prevent SARS-CoV-2 viral replication [39]. However, Tempol has been mainly used in preclinical studies in animal models, and limited data are available regarding its efficacy and safety, particularly in humans. Although it is generally well tolerated in many animal models, exposure to doses much higher than those used in the present study may lead to toxic effects in certain animal models [40]. These uncertainties may represent a limitation for the future use of Tempol as a therapeutic agent in humans.

Our observations suggest that Tempol may also be effective in reducing the progression of frailty in AD patients. The design of our study indicates that the beneficial effect of Tempol is obtained by the administration of a low dose for a long period of time, encompassing a large part of the animal’s lifespan. In fact, Tempol was administered to mice at the age of 3 months, and analysis of platelet function and measurement of frailty index were performed 6 and 15 months later. The duration and design of the study necessitated limiting the administration of a single low dose of Tempol. It would certainly be of interest in the future to extend the study with a dose–response analysis, which could provide more detailed information to optimize the effects on frailty. However, our results are consistent with the notion that prolonged, permanent low-grade inflammation and oxidative stress may trigger and sustain the onset of frailty. They do not indicate that Tempol has an immediate therapeutic effect but demonstrate that a constitutive, long-term prevention of oxidative stress and inflammation is an effective strategy to prevent the development of frailty associated with AD. Although such a prolonged pharmacological approach is unlikely to be easily translated to humans, our results outline how a healthy and correct lifestyle in association with a correct diet rich in anti-oxidant nutrients may be recommended for healthy aging.

It is reasonable that several cellular types are targeted upon administration of Tempol to living mice, and many of them can be responsible for the beneficial effect on the onset of frailty. In this study, we have focused our attention on blood platelets as possible target candidates based on several previous observations pointing to a novel role of these cells in AD development. In addition to their haemostatic functions, platelets also exert pro-inflammatory activity and can trigger ROS-dependent oxidative reactions when exposed to the amyloid peptide Aβ [10, 25]. Platelet hyperactivation has been documented in several AD murine models [28, 29]. Platelets are the major source of Aβ peptides and may contribute to the onset of AD and of CAA both in humans and mice [35]. Since inflammation and oxidative stress have been proposed to predispose to frailty development, platelets appear plausible candidates for regulation also of frailty in the course of AD. In this scenario, our data confirm this hypothesis, as prevention of frailty progression in Tempol-treated APP23 mice is associated with a reduction of platelet reactivity both in terms of aggregation and formation of pro-inflammatory platelet-neutrophil aggregates. Although it is possible that other cells may play a role in mediating the beneficial effect of Tempol, our results recognize a novel important function for blood platelets.

WT mice were used as control in this work, but the effect of Tempol on the progression of frailty and on platelet functions was limited to the APP23 mice. However, we have preliminary evidence that Tempol may have a beneficial effect on the development of frailty also in healthy control animals, suggesting that the advantage of a long-term anti-oxidant treatment may be beneficial for the quality of aging in all individuals. This possibility, however, needs to be more precisely addressed. Because of the complexity and the duration of this study, we have been unable to include additional groups of animals. It would be very interesting, for instance, to investigate whether a long-term therapy with a classical anti-platelet agent, such as aspirin or clopidogrel, may have similar effects to Tempol on frailty development, in order to better appreciate the relative contribution of the pro-thrombotic versus the pro-inflammatory roles of platelets. These investigations, however, clearly require a different, specifically designed study.

This study has some evident limitations, mainly due to the fact that its long duration prevented modifications to the original plan during the 18 months of analysis. Therefore, several analyses that could have strengthened the observations could not be performed. For instance, although our work reports, for the first time, a correlation between the pro-inflammatory actions of platelets and the onset of frailty in the context of AD, the precise molecular mechanisms remain to be clarified. Moreover, the relatively limited number of animals included in the study restricted the possibility of conducting detailed dose–response experiments, as well as the analysis of different platelet antagonists, and reduced the statistical significance of the results. Nevertheless, the relevance of this study lies in the first demonstration that platelet hyperreactivity and pro-inflammatory activity are linked to the onset of frailty in the context of AD, adding an important piece of information to the pathophysiology of both diseases.

In conclusion, the present study provides evidence showing that an anti-oxidant agent able to control platelet reactivity and pro-inflammatory function may improve the quality of aging even in the presence of age-associated disease such as AD and outline the importance of a correct lifestyle for a healthy aging.

## Supplementary Information

Below is the link to the electronic supplementary material.

ESM 1 Supplementary Material 1 (PNG 499 KB)

High Resolution Image (TIF 26.9 MB)

ESM 2 Supplementary Material 2 (PNG 516 KB)

High Resolution Image (TIF 27.0 MB)

ESM 3 Supplementary Material 3 (PNG 398 KB)

High Resolution Image (TIF 26.5 MB)

ESM 4 Supplementary Material 4 (DOCX 16.0 KB)

## Data Availability

Not applicable.
