# Supplementary figures and images for: Platelet hyperreactivity and frailty in a mouse model of Alzheimer’s disease are prevented by anti-oxidant treatment
Source: GeroScience. 2025 Jun 3;48(1):879–96. doi: 10.1007/s11357-025-01710-w (PMC12972370; doi:10.1007/s11357-025-01710-w)

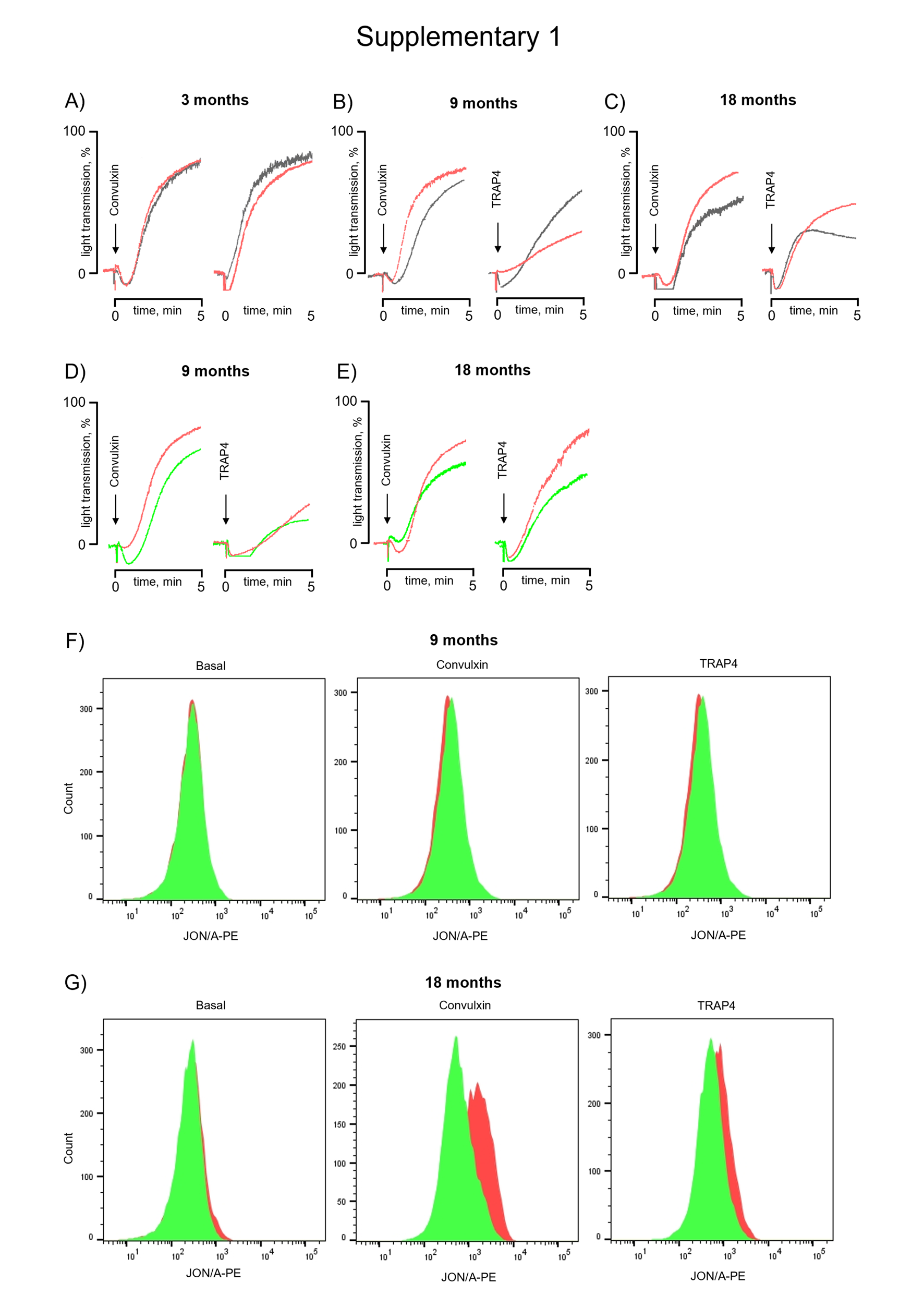

Supplement: Supplementary file 1 — Supplementary Material 1 (PNG 499 KB) [file 11357_2025_1710_Fig5_ESM.png]

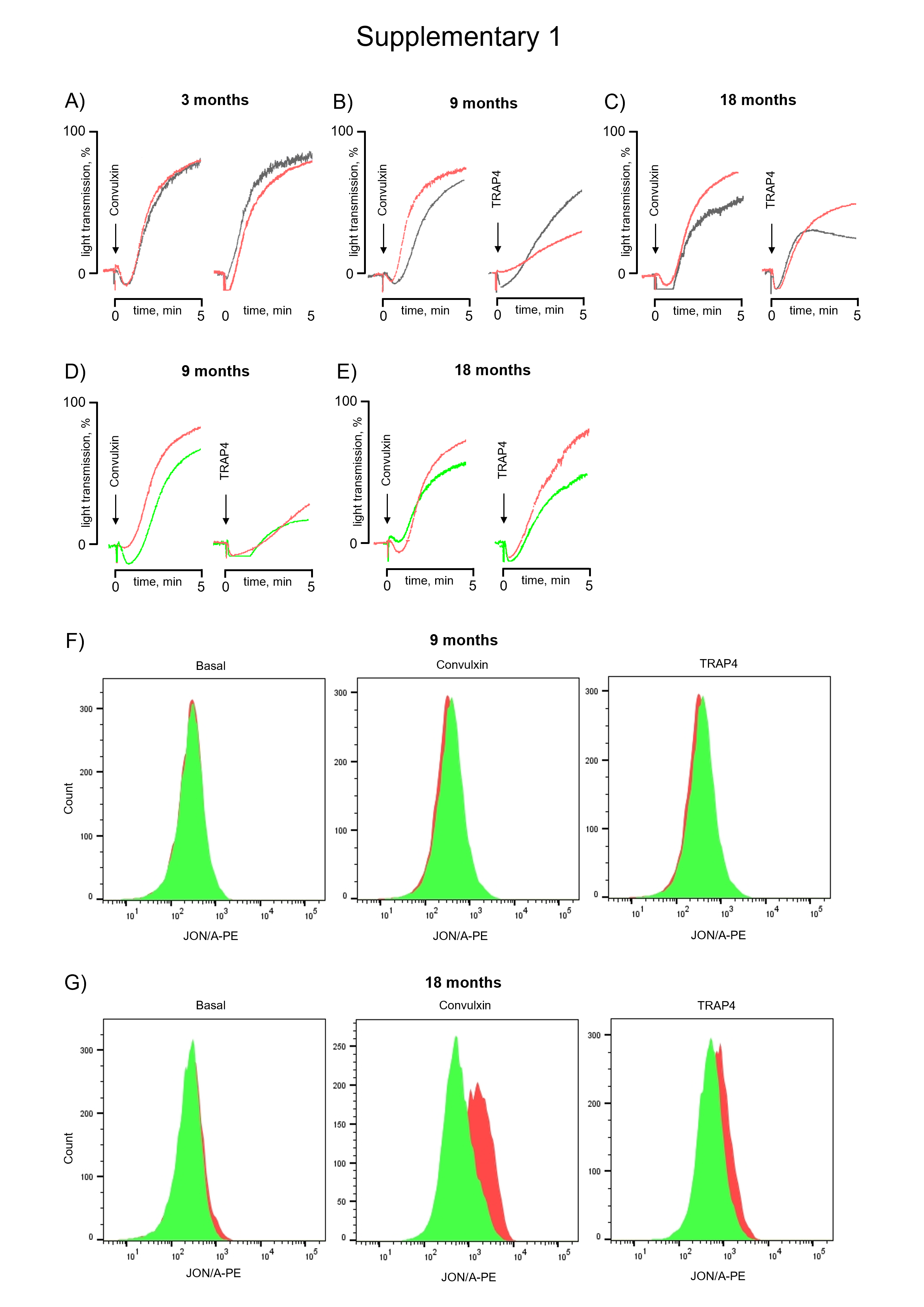

Supplement: Supplementary file 2 — High Resolution Image (TIF 26.9 MB) [file 11357_2025_1710_MOESM1_ESM.tif]

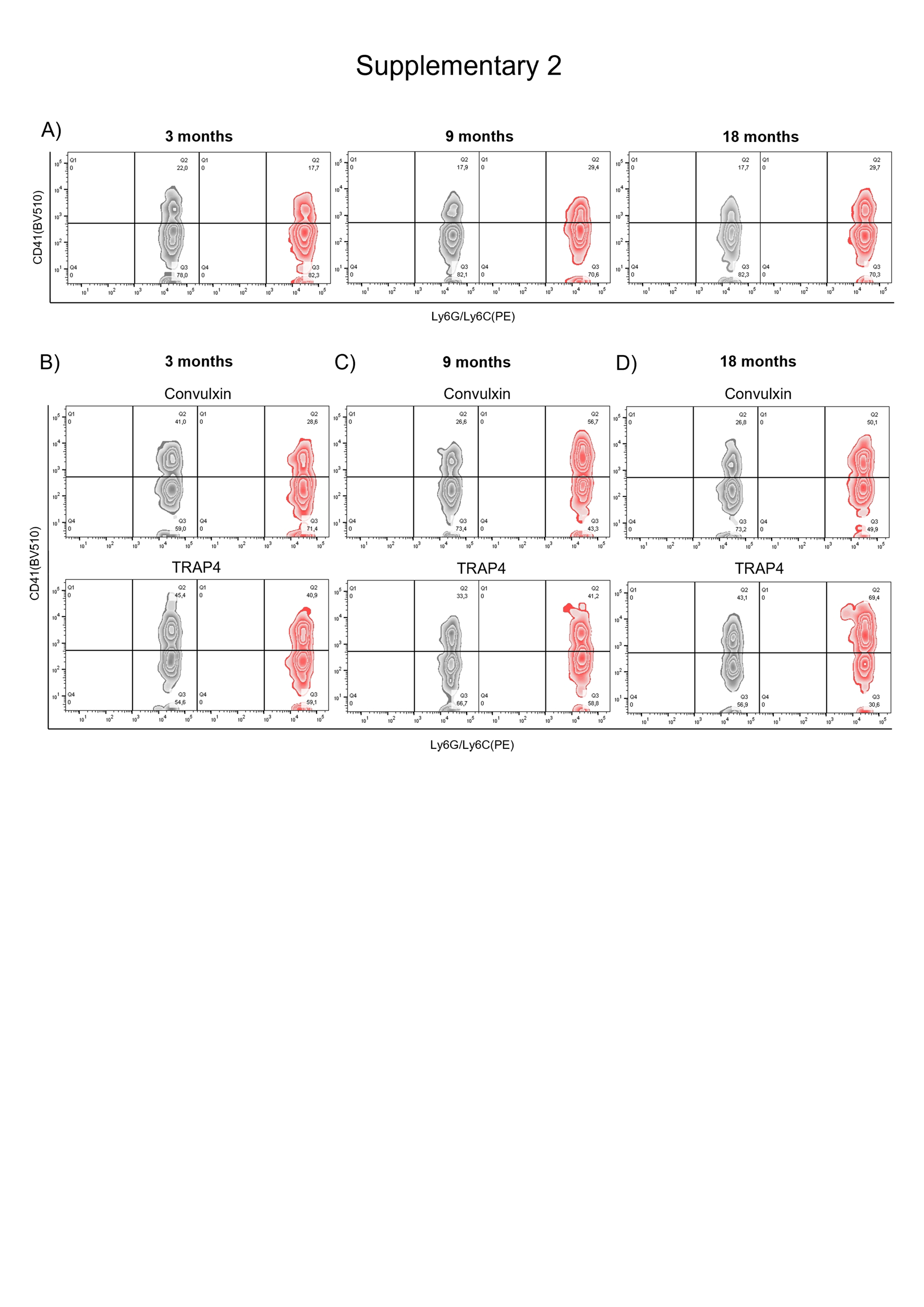

Supplement: Supplementary file 3 — Supplementary Material 2 (PNG 516 KB) [file 11357_2025_1710_Fig6_ESM.png]

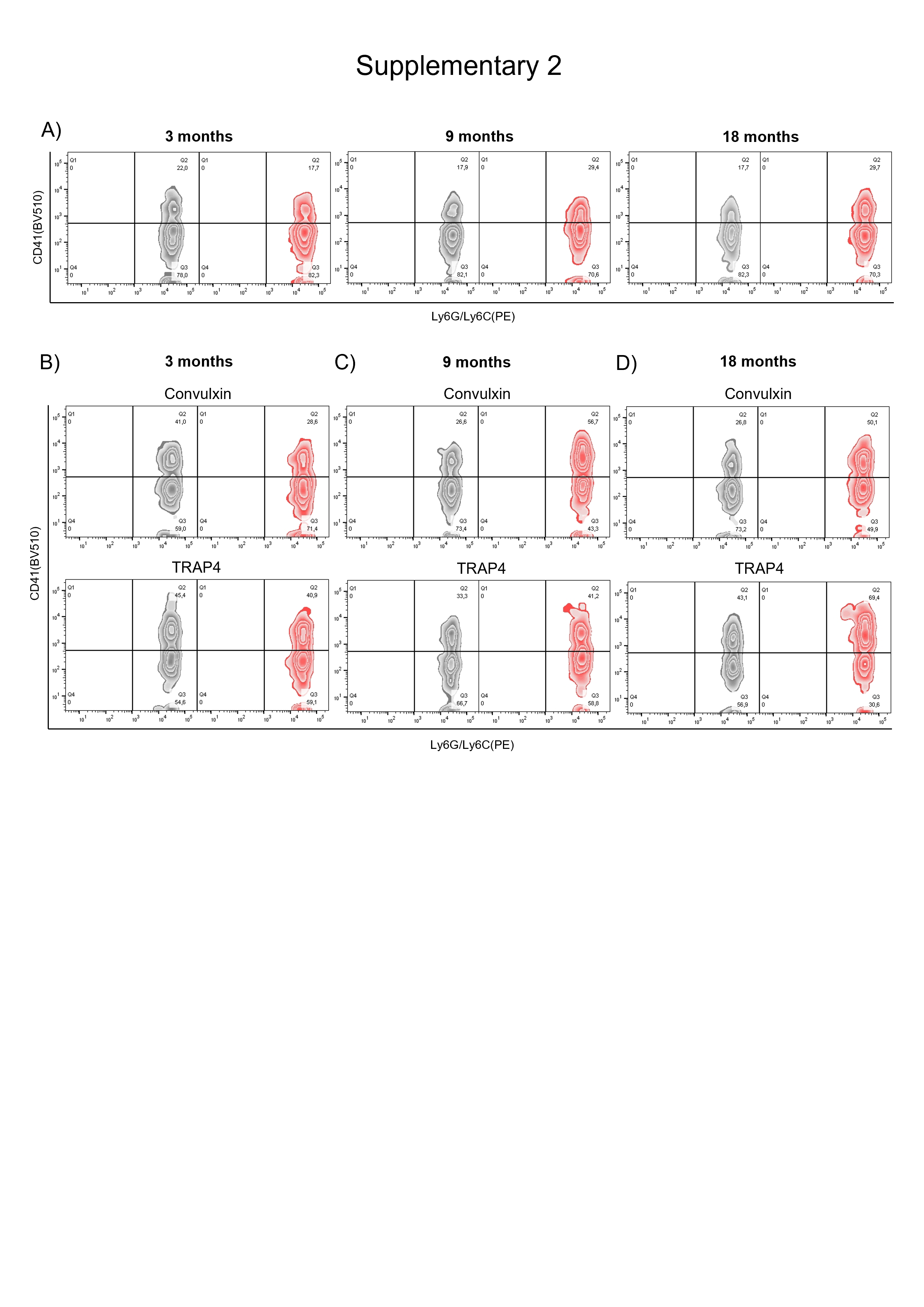

Supplement: Supplementary file 4 — High Resolution Image (TIF 27.0 MB) [file 11357_2025_1710_MOESM2_ESM.tif]

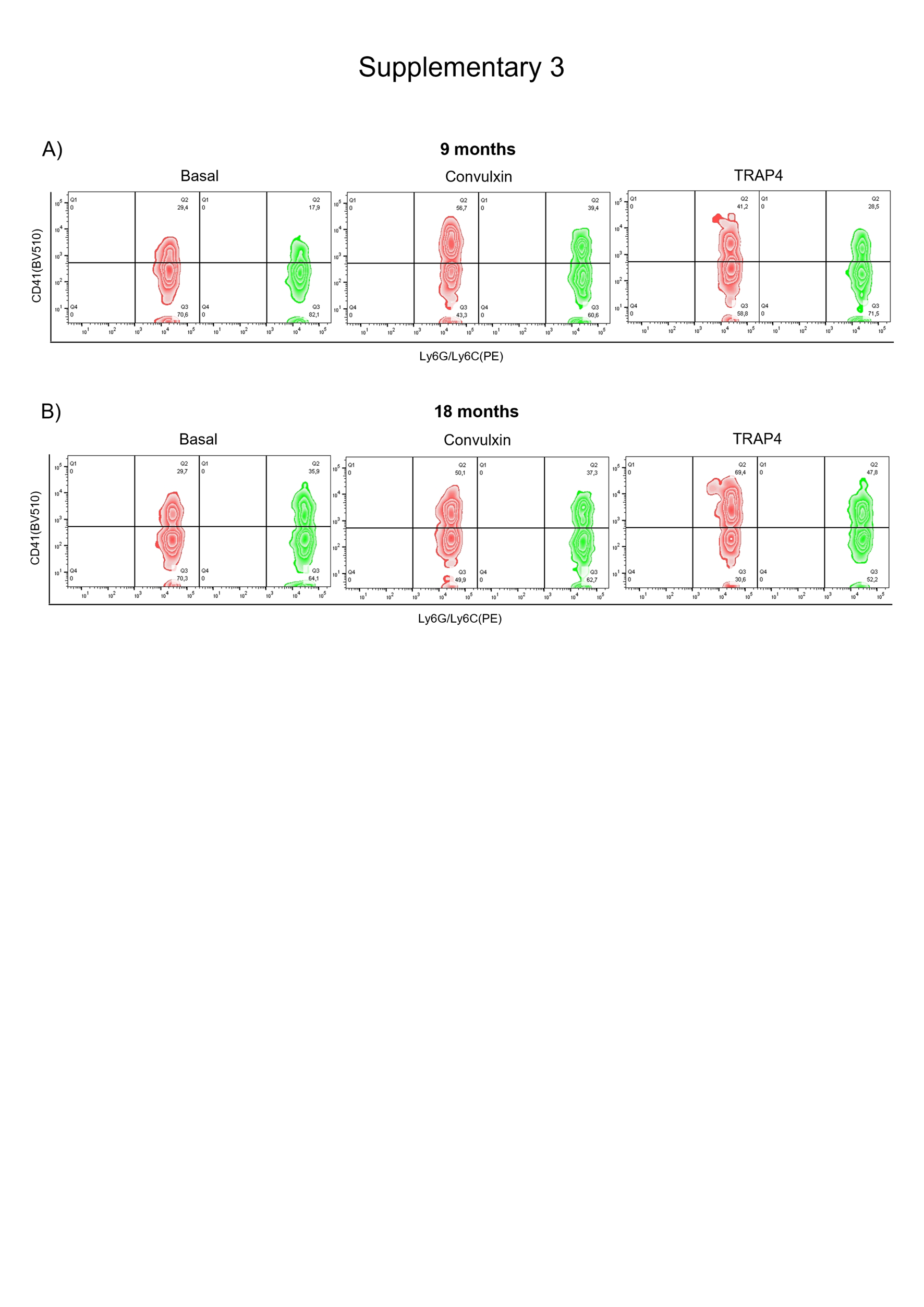

Supplement: Supplementary file 5 — Supplementary Material 3 (PNG 398 KB) [file 11357_2025_1710_Fig7_ESM.png]

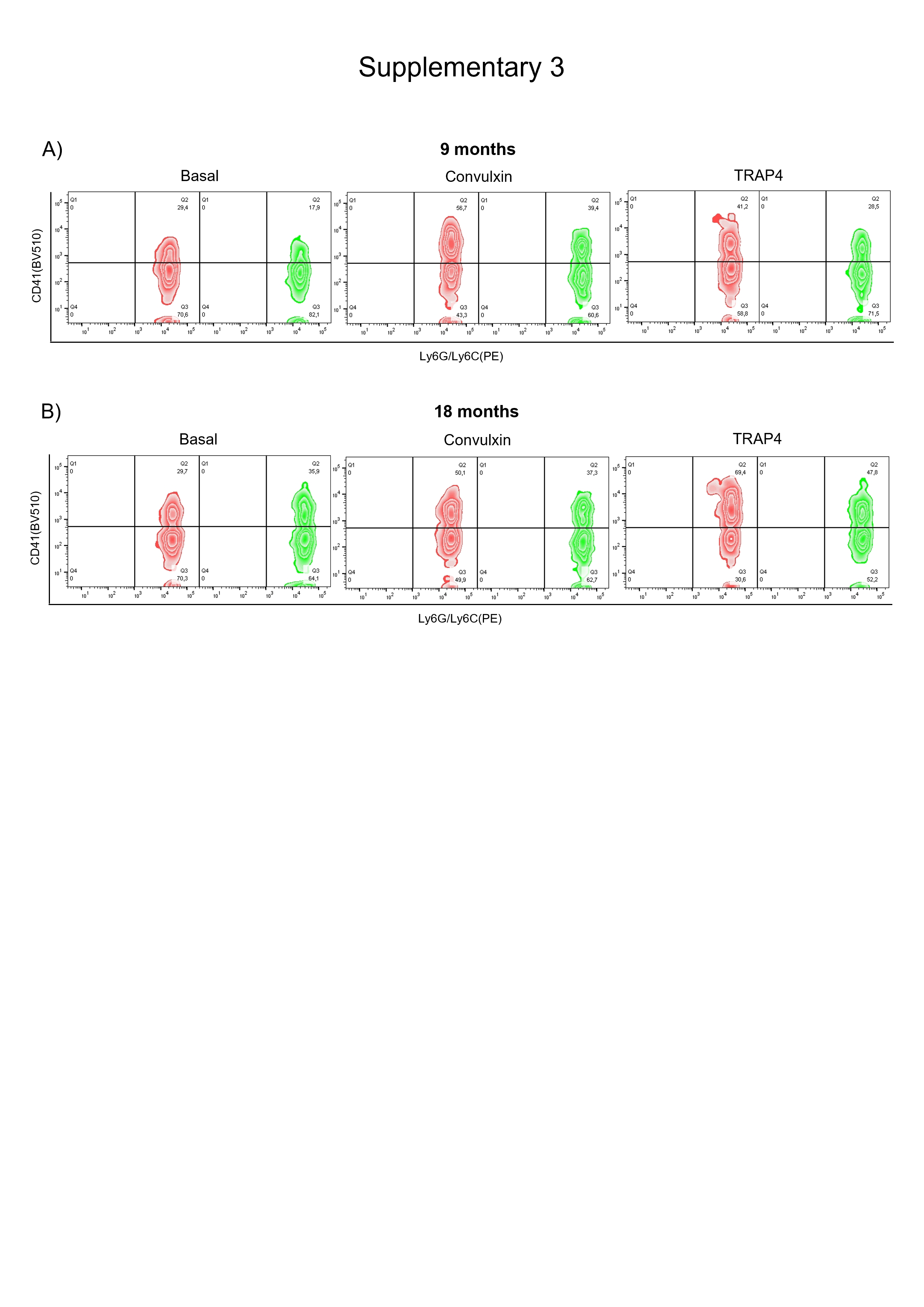

Supplement: Supplementary file 6 — High Resolution Image (TIF 26.5 MB) [file 11357_2025_1710_MOESM3_ESM.tif]
