# Supplementary material for: Platelet hyperreactivity and frailty in a mouse model of Alzheimer’s disease are prevented by anti-oxidant treatment
Source: GeroScience. 2025 Jun 3;48(1):879–96. doi: 10.1007/s11357-025-01710-w (PMC12972370; doi:10.1007/s11357-025-01710-w)
Supplement: Supplementary file 7 — Supplementary Material 4 (DOCX 16.0 KB) [file 11357_2025_1710_MOESM4_ESM.docx]

**SUPPLEMENTARY FIGURE LEGENDS**

**Supplementary Figure 1. Representative aggregation traces and integrin αIIbβ3 activation**

Panels A, B, and C. Representative platelet aggregation traces are shown for WT (grey lines), untreated APP23 (pink lines) and stimulated with convulxin (25-50 ng/ml) or TRAP4 (0,25-0,5 mM). Arrows indicate the addition of agonists. Aggregation was monitored in platelets from mice aged 3, 9, and 18 months, as shown on the top.

Panels D and E. Representative platelet aggregation traces from APP23 mice untreated (pink lines) or treated with Tempol (green line) and stimulated with convulxin (25-50 ng/ml) or TRAP4 (0,25-0,5 mM). Arrows indicate the addition of agonists. Aggregation was monitored in platelets from mice of 9 and 18 months of age, as indicated on the top.

Panels F and G. Flow cytometry analysis of integrin αIIbβ3 activation (JON/A binding) in whole blood from untreated APP23 (pink peaks) and Tempol-treated APP23 (green peaks) mice, stimulated with convulxin (25-50 ng/ml) or TRAP4 (0.25-0.5 mM) for 10 minutes, as indicated.

**Supplementary Figure 2.** **Representative plots of PNA formation in WT and APP23 mice under basal and stimulated conditions**

Representative PNA zebra plots of wild type (grey) and untreated APP23 (pink) mice are shown. Panel A displays the constitutive PNA detectable in mice of the two genotypes at 3, 9, and 18 months of age, as indicated above. Panels B, C, and D depict PNA formation upon stimulation of platelets with convulxin (25-50 ng/ml) or TRAP (0,25-0,5 mM) in the two genotypes at 3, 9, and 18 months of age.

**Supplementary Figure 3.** **Representative plots of PNA formation in APP23 mice at different ages after Tempol treatment**

Representative PNA zebra plots of untreated APP23 (pink) and Tempol-treated APP23 (green) mice. Panel (A) presents PNA formation in 9-months-old mice under basal conditions and after platelet stimulation with convulxin (25-50 ng/ml) or TRAP (0,25-0,5 mM). Panel B illustrates PNA formation in 18-month-old mice under basal conditions and upon stimulation with convulxin (25-50 ng/ml) or TRAP4 (0,25-0,5 mM).
